# Chinese American Immigrant Parents' Socialization of Emotions in Bilingual Bicultural Preschool Children

**DOI:** 10.3389/fpsyg.2021.642417

**Published:** 2021-07-30

**Authors:** Shirley Huang, Pui Fong Kan

**Affiliations:** Department of Speech, Language, & Hearing Sciences, University of Colorado Boulder, Boulder, CO, United States

**Keywords:** bilingualism, emotion, immigrants, sociocultural, Chinese

## Abstract

The purpose of this study was to examine Cantonese-speaking Chinese American immigrant parents' socialization of emotions in bilingual bicultural preschool children, using a combination of a parent questionnaire and parent language samples from emotion-elicited storytelling tasks. Sixteen Cantonese-speaking parents and their children participated in this study. Children were sequential bilinguals who were exposed to Cantonese (L1) at home since birth, and then learned English (L2) at school. The Chinese parent questionnaire examined parents' emotion talk in the home, as well as the child's dual language background and language distribution. Parents' language samples in Cantonese were collected from three parent-child storytelling tasks that each elicited a different type of negative emotion (sad, angry, scared). Results from the parent questionnaire and the parent language samples were analyzed using quantitative and qualitative methods. In the parent questionnaire, correlation analysis revealed that parents' use of guilt emotions was not associated with any of the other emotion words, suggesting that parents may not talk about guilt as frequently as the other emotions. Results from the parents' language samples showed no significant differences between parents' number of emotion words and emotion explanations across the storytelling tasks, suggesting that parents used negative emotion words similarly across all three books. Further qualitative analysis between the parent questionnaire and the language samples revealed patterns in the way parents use Chinese emotion words with their children. Findings illustrate how the combined use of a parent questionnaire and parent language samples offer complementary information to provide a more comprehensive understanding about Chinese American immigrant parents' socialization of emotions.

## Introduction

One of the earliest contexts in which children first learn emotion words is through parent-child interactions in the home environment. Parental emotion-related socialization behaviors (ERSBs), including labeling and discussion of emotions, shape how children experience, understand, or express emotions (Eisenberg et al., 1998). Many cultural, social, and language factors could influence parents' socialization of emotions with their children in the home (e.g., Keltner and Haidt, 1999; van Kleef et al., 2016). For instance, cross-cultural studies have shown that media, magazines, and books tend to display culturally appropriate emotion behaviors (e.g., facial expressions, tone, body language) (Tsai et al., 2007; Wege et al., 2014), social practices in high vs. low arousal activities reflect differences in emotional intensity preferences (Tsai, 2007; Lim, 2016), and differences in the emotion lexicon across languages suggest varied emotional experiences and perspectives (Wong and Tsai, 2007; Pavlenko, 2008). This is especially important for immigrant children or children from immigrant families in the United States who are exposed to their home language (L1) (e.g., Cantonese, Spanish, Japanese) since birth and later learn English (L2) in a school setting. Despite increasing literature on the important role parents have in children's emotion language development, there are relatively fewer studies that examine parents' socialization of emotions in bilingual bicultural children in the home environment.

Many newly arrived immigrant families may live in urban areas (e.g., Chinatown) with a high concentration of culturally specific establishments such as grocery stores, restaurants, or community centers, and where the residents may predominantly speak different dialects of the same language or share a similar cultural background (Tsui, 2010). These communities are likely to help preserve and reinforce the home culture, language, and social practices, which may also contribute to how parents socialize emotions with their bilingual child in the home. Since bilingual bicultural children are first exposed to emotion words in a home language that is different from English and in a sociocultural context that is different from the mainstream American classroom setting, studying early parent socialization of emotions beginning in the home may reveal better ways to support bilingual children when they enter the classroom setting.

This study examined Chinese American immigrant parents' emotion talk in bilingual bicultural preschool children who were exposed to Cantonese (L1) at home from birth and started to learn English (L2) in preschool. Parents' socialization of emotions with their children has been studied using parent questionnaires to collect developmental norms on emotion words (i.e., Ridgeway et al., 1985; Baron-Cohen et al., 2010), examine parent-child conversations related to emotions (Mazzone et al., 2017), and gather information about parents' beliefs about emotions (Halberstadt et al., 2013). Additionally, parent language sampling is an informative measure that could reveal how parents use emotion labels and explain emotions to their children in a more natural context (Cervantes and Callahan, 1998; Aznar and Tenebaum, 2013). Despite a growing number of tools and measures to assess emotion language skills in children, there are relatively few measures for bilingual children (Humphrey et al., 2011), and even fewer measures exist to evaluate parents' emotion talk in bilingual children in the home. The combined use of a parent questionnaire and parent language samples can gather information about the child's emotion language environment at home and capture parents' specific emotion words used with their child, potentially providing more comprehensive information regarding parents' socialization of emotions.

The main goal of this study was to examine parents' emotion talk with their child in the home language because it is the first language in which the child is exposed to emotion words. The second goal was to explore the use of a parent questionnaire and language samples to gather more holistic information about parents' emotion talk. A comprehensive understanding of bilingual bicultural children's emotion language in the home environment has important implications for informing clinicians and educators about developing more culturally-linguistically appropriate therapy and teaching activities that align with what parents are already doing in the home to support their child's learning needs.

### Parents' Emotion Talk in the Home Environment

One of the earliest contexts in which children are first exposed to emotion language is in their home environment through parent socialization. Eisenberg et al. (1998) identified three types of parental emotion-related socialization behaviors (ERSBs) that influence and promote children's emotion language skills, including parental reactions to children's emotions, discussion of emotions, and expression of emotions. Most of the research on parental ERSBs was predominantly done on White American monolingual English-speaking populations (e.g., Denham and Kochanoff, 2002; Eisenberg et al., 2005), and less is understood about the socialization of emotions in parents from minority cultural and language backgrounds. In the present study, we focus specifically on parents' discussion and expression of emotions in the home language with their bilingual bicultural preschool children.

Emotion understanding and expression are shaped by our interactions with other people within a social cultural context (e.g., Keltner and Haidt, 1999; van Kleef et al., 2016). Through engagement in and exposure to social and cultural practices, people gradually internalize their culture's notion of emotions (Tsai, 2007; Wege et al., 2014; Lim, 2016). Many studies have shown that social, cultural, and language factors influence parents' socialization of emotions in bilingual children in the home (see review in Halle et al., 2014). For example, greater exposure to and frequent use of the home language with parents can foster emotion understanding and well-being in bilingual Singapore preschool children (Sun et al., 2018; Sun, 2019). In another example, Aznar and Tenebaum (2013) found that in Spanish-English bilingual children maternal emotion talk, but not paternal, was related to children's emotion understanding, which was consistent with Spain's traditional gender-prescribed caregiver norms and expectations. Moreover, emotion words may vary across languages and cultures such that some emotion words used in one language may not have an equivalent translation in another language or may not evoke the same affective state in a different sociocultural context (Keltner and Haidt, 1999; Pavlenko, 2008). Therefore, cross-linguistic differences suggest different emotional experiences and perspectives in each language, which may in turn influence how parents talk about emotions with their children. Indeed, Chen et al. (2012) showed that in a multilingual family context, which language parents use in the home to talk about emotions contributes to children's own emotion understanding, expression, and regulation of emotions in each language.

For many immigrant parents in the United States, they may continue to use the home language when communicating with their children and other family members in the home, which may influence how parents socialize emotions with their child. Many sequential bilingual children first learn emotion language in their home language with their parents at home, but then they transition to an English-speaking school environment and begin learning emotions in a second language with their peers and teachers. Unlike monolingual children, bilingual children may consequently experience a mismatch between the social, cultural, and language factors in the home context and those in a main-stream American English-speaking school context (e.g., Heath, 1982; Commins, 1989; Baker and Páez, 2018). Given the dual cultural and linguistic environments in which young bilingual children grow up, we would expect that parents' socialization of emotions is qualitatively different than that in parents of monolingual children.

### Cultural Considerations of Emotions in Chinese American Immigrant Families

The current research focused on parents' socialization of emotions in Chinese American immigrant families whose children speak Cantonese as a home language and use English in a school context. Chinese American parents' unique exposure to different sets of values and social norms between the home and host culture may influence bilingual bicultural children's emotion understanding and expression (Tsai, 2007; Chentsova-Dutton and Tsai, 2010; Chen et al., 2015). Emotions are embedded in larger cultural institutions and social practices which shapes one's emotional expression, interpretation, and experiences (e.g., Keltner and Haidt, 1999). For example, a study examining emotion displays in preschool children's storybooks in Romania, Turkey, and the United States revealed cultural differences in how frequently powerful (e.g., anger) and powerless (e.g., sad) negative emotions are displayed, suggesting that children are exposed to culture-specific emotion norms and values. Similarly, we see differences in emotion display in Chinese and American societies. Differences in display of emotion intensity in media, magazines, and movies (e.g., open vs. closed smiles) and activity-seeking preferences (e.g., mountain biking vs. picnicking) reflect the society's value and use of high-arousal (e.g., excitement, enthusiasm) and low-arousal (e.g., peace, calm) emotion intensity words (Tsai, 2007; Lim, 2016).

Additionally, eastern countries like China value a collectivist society and place greater emphasis on behaviors that directly impact group harmony, while western countries like the United States value an individualistic society and encourage agency in one's emotions and ideas to promote autonomy (e.g., Wong and Tsai, 2007). Since guilt emotions play a significant role in signaling whether one has violated the moral standards and norms in their society (Lagattuta and Thompson, 2007), guilt is highly valued in collectivist societies (Yik, 2010). Several cross-cultural studies have documented cultural differences in guilt between western and eastern countries (e.g., Bedford, 2004; Wong and Tsai, 2007). Bedford (2004) interviewed adults in Taiwan and identified three main types of guilt words and their meanings: failure in one's personal responsibilities (*nei jiu*), moral transgression (*zui e gan*), and breaking a law or rule (*fan zui gan*). These subtypes of emotion words for guilt in Chinese refer to different behaviors and intensity levels that are indistinguishable in English (Bedford, 2004).

To date, there are no studies that directly examine Chinese parents' use of guilt emotion words with their child in the home context. However, one longitudinal study examined Chinese parents' socialization of shame with their preschool child from age 2.5–4 years old (Fung, 1999). Nine Taiwanese families living in Taiwan, who spoke Mandarin and Taiwanese, were systematically videotaped and observed in their homes every 3 months for 2 h each. A total of 140 h of videotaped family interactions were transcribed and coded for events of shame, including labeling shame (“shame on you”), gestures and body language related to shame, and idiomatic expressions for shame. Results showed an average rate of 2.5 events of shame per hour between parents and their child, and socialization of shame occurred as early as 2.5 years of age. Although shame and guilt are considered distinct emotions, they are both part of the self-conscious emotions' family (see Tangney et al., 1996). Findings from Fung (1999) may provide preliminary information on how Chinese parents socialize self-conscious emotions and whether this socialization pattern may be seen in guilt too. Exploring the frequency and use of emotion words, including self-conscious emotions like guilt, may contribute to our understanding of Chinese American immigrant parents' socialization of emotions in the home.

Even among negative valence emotion words (e.g., sad, angry, scared), there are cultural variations in the valuation, expression, and behavioral consequences of each type. Fivush and Wang (2005) studied mothers living in China and in the United States during a reminiscing story task with their 3-year-old child to examine whether there were cross-cultural differences in mothers' use of negative emotion words. They found that Chinese mothers discussed more angry emotions with their children, while American mothers discussed sadness more frequently. Their findings suggested that Chinese mothers may be concerned with helping their children learn appropriate reactions to and regulation of angry emotions to maintain social harmony (Fivush and Wang, 2005). In our study, parents are Chinese American immigrants whose children are bilingual and bicultural, and so given their unique dual sociocultural exposure, it is worth exploring parents' use of different types of emotion words, including negative emotion words.

Chinese American immigrant parents may show different socialization patterns with their child compared to Chinese mothers and American mothers, and that may be associated with different sets of social constructs and parents' cultural orientation (Tao et al., 2013; Chen et al., 2015; Curtis et al., 2020). Chinese American parents may be in the process of adapting to the mainstream American culture (acculturation) while maintaining practices and beliefs in their Chinese culture (enculturation) (Tao et al., 2013). Chen et al. (2015) examined Chinese American immigrant parents' orientation to Chinese and American cultures and how often they express their emotions using self-reported questionnaires. They found that parents' orientation to Chinese culture such as Chinese media use (e.g., TV shows, movies) was negatively associated with emotional expressivity, whereas parents' American orientation, including English language proficiency and American media use, was associated with greater emotional expressivity. They conclude that varying levels of engagement in the host and home culture can result in differences in emotional expression. Their findings are consistent with other studies which suggest that even among Chinese American immigrant populations there is a lot of heterogeneity in the home environment, socioeconomic status, language background and proficiency, and parenting socialization behaviors as they relate to children's emotion outcomes (e.g., Han and Huang, 2010; Curtis et al., 2020). In our study, parents are Chinese American immigrants who speak Cantonese as the home language with limited English proficiency. Understanding the nuances in Chinese American immigrant families' cultural orientations is important in gaining a holistic understanding and making an informed interpretation of parents' emotion talk with their child.

### Combined Use of a Parent Questionnaire and Parent Language Samples

In clinical and educational settings, parent questionnaires not only reliably estimate bilingual children's dual language skills (e.g., Paradis, 2010; Thordardottir, 2011; Cheung et al., 2019), but they also provide a functional perspective of the home environment that may not be captured in more narrow assessment measures (e.g., Gutierrez-Clellen and Kreiter, 2003; Ebert, 2017; Byers-Heinlein et al., 2018). Of particular interest in this study is the use of a parent questionnaire to examine parents' emotion talk in Cantonese-English bilingual children.

For bilingual children, the use of a parent questionnaire about emotions can serve dual purposes: not only can it collect information about their emotion language in the home environment, but it also puts the information into the larger context of the bilingual child's dual language and cultural profile (e.g., Halle et al., 2014). Indeed, several studies have used parent questionnaires as a broad measure to collect information on emotion language skills in Chinese-English bilingual children, as well as gather their demographic information and language history to support interpretation of the study findings (e.g., Sun et al., 2018; Sun, 2019). Sun et al. (2018) used parent and teacher questionnaires, along with standardized vocabulary and cognitive tests, to examine the relationship between bilingual language experience and social-emotional behavioral skills in 805 Singaporean preschool children. The parent questionnaire collected information about children's dual language exposure and use in the home, while the teacher questionnaire reported the children's social-emotional and behavioral strengths and difficulties in the classroom. They found that greater bilingual language proficiency, as reported on the parent questionnaire, was significantly related to better social-emotional and behavioral skills (Sun et al., 2018). Although parent questionnaires can collect information about children's emotion language experiences in the home, what emotion words parents use with their child and in which contexts may not be captured accurately or may not be captured at all. Therefore, it is possible that using more narrow measures such as parent language samples, elicited through a parent-child storytelling context, can collect more fine-grained information about bilingual children's emotion language input.

Many studies have used play activities and storytelling contexts to elicit emotion language and collect language samples from parents. Cervantes and Callahan (1998) examined 84 children (2–4 years old) and their mothers during mother-child play and storytelling activities and coded the number of emotion labels, causes, and explanations. Similar methodology of collecting parent language samples and coding emotions has been implemented on other languages, including Chinese (Wang et al., 2000) and Spanish (Aznar and Tenebaum, 2013), to examine cross-cultural differences in emotion language. For example, Wang et al. (2000) examined a total of 41 White-American and Chinese mothers living in Boston and Beijing, respectively, and their 3-year-old children during two mother-child conversations tasks: sharing memories and telling a story from a picture book. They coded mothers' affect statements and questions in their language samples and found gender differences, such that mothers of boys discussed more positive emotions when sharing memories, while mothers of girls made more comments on the story character's negative emotions. These findings suggest that parent language samples can reveal fine-grain information about children's exposure to types of emotion words from their parents in a more natural context. However, they did not collect the child's background information and emotion language skills in the home context. Language samples only present a snapshot of parents' emotion language use within a specific storytelling context, and they do not reveal a wider range of emotion words that parents may use at home—words that may have been identified in a broader measure such as a parent checklist or questionnaire.

Relevant to our study, Curtis et al. (2020) used a combination of parent language samples and parent questionnaires to examine the relationship between Chinese American immigrant parents' emotion talk and their school-age (6–9 years old) children's emotion skills (*n* = 258). While the parent language samples analyzed parents' use of emotion words, questions, and elaborations, the parent questionnaires focused on their child's emotion regulation and social emotional behavioral skills, but not the parents' emotion talk in the home. Results showed that parents' emotion talk from the language samples was associated with children's higher emotional regulation and socioemotional skills. In our study, the questionnaire collects information on parents' emotion talk in the home, as well as the children's distributed dual language input. Supplementing parents' language samples with parent questionnaires could provide more holistic information to understand bilingual bicultural children's early socialization of emotions in the home.

### The Current Study

The purpose of this study was to examine Chinese American immigrant parents' socialization of emotions in children who are exposed to Cantonese (L1) at home and learn English (L2) at school, using a parent questionnaire and parent language samples to gather holistic information about bilingual bicultural children's home environment. Despite increasing attention to emotion language skills in young children, there are few measures for culturally-linguistically diverse groups of children (Humphrey et al., 2011). The use of both a parent questionnaire (broad measure) and parent language samples (narrow measure) has potential to advance our understanding of parents' emotion talk in bilingual bicultural children.

Building upon and adapting from existing parent questionnaires (e.g., Mazzone et al., 2017; Cheung et al., 2019), we implemented a Chinese parent questionnaire to broadly capture immigrant parents' socialization of emotions in the home environment. Additionally, following previous studies (e.g., Aznar and Tenebaum, 2013; Curtis et al., 2020), we implemented parent storytelling tasks and each book elicited a different type of negative emotion word (sad, angry, scared). Parent language that occurs during a storytelling context is more linguistically diverse and syntactically complex than parent language that occurs in play interactions (e.g., Demir-Lira et al., 2018). This study addressed the following research questions:

1. How do Chinese American immigrant parents socialize emotions as measured by a parent questionnaire (broad measure)?
2. How do Chinese American immigrant parents socialize emotions as measured by parent language samples (narrow measure)?
3. What does the combined use of the parent questionnaire and the language samples tell us about how parents use Chinese emotion words with their children?

Since the parents in our study are Chinese American immigrants, who immigrated from China and still use Chinese as their dominant language at home with their child, it is likely that parents' socialization of emotions would be similar to what we see in Chinese society (e.g., Keltner and Haidt, 1999; Tsai, 2007; Lim, 2016). We hypothesized that the parent questionnaire would show that parents may talk about certain emotion words more frequently than others. In particular, we anticipated that parents would talk about self-conscious emotions like guilt frequently with their child, as is seen in previous studies examining Chinese parents and adults (e.g., Fung, 1999; Bedford, 2004; Wong and Tsai, 2007). For the parent language samples, we hypothesized that parents may use more angry emotion words compared to the other types of negative emotion words because Chinese parents may be concerned with helping their child regulate anger to maintain social harmony (Fivush and Wang, 2005). Lastly, since parent questionnaires and language samples each provide different information about parents' emotion talk (e.g., Cervantes and Callahan, 1998; Sun et al., 2018; Sun, 2019; Curtis et al., 2020), we expected that the combined use of both may reveal patterns in which emotion words parents use with their children in different contexts.

## Method

### Participants

A total of sixteen Chinese American immigrant parents and their Cantonese-English bilingual preschool children (mean = 4;1 years; *SD* = 0.55, range = 3;4–5;0 years,) were recruited though the Kai Ming Head Start preschools in San Francisco, California. Kai Ming Head Start is a non-profit, government-funded program which provides preschool services to children from low-income families based on the federal poverty guidelines. The children and families who participated in this study come from low socioeconomic backgrounds. Most parents reported having a high school education, three parents had a college education, and one parent had a primary school education. Parents were native Cantonese-speakers with limited English proficiency. Children were sequential bilinguals, who were exposed to Cantonese at birth (L1) and then started to learn English as a second language (L2) in the school setting. Parents did not report concerns with speech, language, hearing, or learning. Table 1 summarizes the characteristics of this group of participants. The participants had attended the Head Start program for an average of 12.56 months (*SD* = 7.80, range = 2–28 months). Children's receptive and expressive vocabulary skills in Cantonese and in English were measured on two separate testing sessions using the Cantonese-English bilingual vocabulary test developed for this population. The order of the language was counter balanced. The scores from the Cantonese and English receptive and expressive vocabulary measures are consistent with previous studies (see Kan et al., 2020). There were no significant differences between children's Cantonese and English receptive and expressive vocabulary skills in our study.

**Table 1:** Summary of children's demographic information and vocabulary scores.

	**Mean**	***SD***	**Range**
Age (in months)	49.20	6.60	40–60
Time in preschool (in months)	12.56	7.80	2–28
Picture identification Cantonese (% correct)	74.38	11.12	44–86
Picture identification English (% correct)	64.51	16.69	26–89
Picture naming Cantonese (% correct)	44.60	17.55	10–71
Picture naming English (% correct)	42.29	24.05	3–83

### Language Distribution in the Home

Parents reported the child's language background including which languages are used in the home and the amount of language input from each family member in the home. Each household varied in the number of family members. All the parents reported Cantonese as the primary language used in the home by all family members. All the parents who reported living with their grandmother and/or grandfather reported that the grandparents spoke 100% Cantonese. The majority of the mothers and fathers reported using 80–100% Cantonese. Only one set of parents reported using 80% English in the home. However, they also reported that both grandparents live in the home and only speak Cantonese, so it is likely the child is exposed to more Cantonese than English in the home. Parents reported that the younger siblings spoke 100% Cantonese at home. However, the older siblings used an increasing amount of English in the home, ranging from 20 to 100% English.

### Home Storytelling Activities

In addition to the child's demographic information and language distribution in the home, the first half of the questionnaire collected information on home storytelling activities to understand the context in which the child is exposed to emotion words. The questionnaire was adapted from a previous questionnaire about parent language input and home activities in Cantonese-English bilingual children (Cheung et al., 2019). Parents reported the hours spent on different story time activities (i.e., reading stories, telling stories, or watching shows) and the percentage of input in each language for each activity. See Appendix A in Supplementary Material for a sample of the questionnaire about storytelling activities. All the parents reported that they engaged in the following storytelling activities with their child: reading stories (with books), telling stories aloud (without books), and watching TV or movies. Although the majority of the participants reported that Cantonese was the primary language used during home activities, there was variation in the amount of L1 and L2 use across activities. Fifty to 62.50% of parents reported that they use 100% Cantonese when reading stories and telling stories aloud with their child. Watching TV or movies comprised the greatest amount of L2 by families.

### Parent Questionnaire on Emotion Language Experiences

The second half of the questionnaire focused on the child's emotion language input in the home. Parents reported which language the parent and the child feel comfortable using when discussing emotions. Next, parents used a Likert rating scale to rate how frequently they explained and labeled emotions with their child in the past 2 weeks (e.g., 1–2 times, 3–4 times). For example, one question asked, “When my child asked me questions about someone being sad, we talked about why that person was sad.” The Likert rating scale portion of the questionnaire was adapted from a previous questionnaire about parent-child conversations about emotions (Mazzone et al., 2017). See Appendix B in Supplementary Material for a sample of the questionnaire about the parent and child's emotion language choice and the Likert rating scale.

The questionnaire also included an emotion word checklist to identify the range of emotion words that parents use with their child. See Appendix C in Supplementary Material for the emotion checklist from the questionnaire. The checklist included five categories of emotions (i.e., Happy, Sad, Angry, Guilt, and Scared), and each category had 9–12 emotion words for parents to select. There was a total of 51 emotion words on the checklist for parents to choose from. Parents had the opportunity to add in additional emotion words that they use with their child in each category. Only two parents reported an additional emotion word in the angry category. However, neither word counted as an emotion word according to our coding criteria, and therefore were not included in the analysis. To date, there are no standardized norms on Cantonese-English bilingual children's emotion word development. Therefore, the emotion words in the checklist were selected based on previously normed data on the comprehension and production of emotion words in monolingual English-speaking children (i.e., Ridgeway et al., 1985; Baron-Cohen et al., 2010) and monolingual Chinese-speaking children (i.e., Li and Yu, 2015). Additionally, the emotion words were selected in consultation with a native Cantonese-speaking research assistant to ensure that the words were culturally, linguistically, and developmentally appropriate for this study's population.

### Parent-Child Emotion Elicitation Storytelling Task

Three wordless picture books were used in this study: (1) “Alexander and the Terrible, Horrible, No Good, Very Bad Day” (Viorst and Cruz, 1977), (2) “There's a Witch Under the Stairs” (Smith, 1991), and (3) “Llama, Llama Misses Mama” (Dewdney, 2009). These books were selected because they have images that depict clear facial expressions that represent basic emotions, have a clear storyline even when the words were removed, and were not familiar to the parents in this study population. Each book elicited a different type of negative emotion word based on the storyline. The “Alexander and the Terrible, Horrible, No Good, Very Bad Day” book, which was about a young boy who was in a bad mood throughout the day, elicited angry emotions. The “There's a Witch Under the Stairs” book, which was about a young girl who attempts to get rid of a witch living under her stairs, elicited scared emotions. The “Llama, Llama Misses Mama” book, which was about a young llama's first day of preschool who is missing his mom, elicited sad emotions. We sought to examine Chinese American immigrant parents' socialization of different types of negative emotion words and whether they may use one type of negative emotion word more often than another within the story context. The books were modified by removing the words from the books and translating the title into Chinese characters. When modifying the books, we kept the three books similar in page length (18–25 pages), without compromising the story flow. All the books were printed on 8 $\frac{1}{2}$” × 11” standard paper size and placed in plastic protection sheets. Each page in the book provided the opportunity to elicit at least one emotion word.

Following the storytelling task, parents completed a brief emotion check measure in Cantonese to confirm that they recognized all the emotions in the storybooks. There were 26 test items in the emotion check measure. The test contained pictures of characters from each book expressing an emotion (e.g., happy, angry, etc.). Parents were asked to label the image with an emotion word. Synonyms of the emotion word were also considered correct (e.g., excited for happy). Scores ≥75% accuracy were considered passing. All the parents scored 75% or greater and passed.

### Procedures

This study employed a cross-sectional design to examine parents' socialization of emotions in sequential Cantonese-English bilingual bicultural preschool children. Two measures were used to examine parents' emotion talk: (1) a Chinese parent questionnaire on children's emotion language experiences (broad measure) and (2) a parent-child emotion elicitation storytelling task (narrow measure) in Cantonese. Individual testing sessions were conducted in a quiet room at the Kai Ming Head Start preschool site and lasted ~90 min. Research assistants who conducted the testing were native speakers of Cantonese and fluent in English. First, parents completed the brief questionnaire in Chinese. The questionnaire took ~10–12 min to complete. Next, parents participated in three emotion elicitation storytelling tasks with their child from wordless picture books. The books intended to elicit emotion words, but research assistants did not tell parents to use emotion words. Parents were asked to tell a story in Cantonese to their child the way they normally would at home. Parents were given several minutes to review the books before telling the story to their child. Parents were asked to tell a story to their child in Cantonese because it is their proficient language and because it is the first language in which the child is exposed to emotion words. Each book elicited a different type of negative emotion word (e.g., sad, anger, scared). Each story was ~10–15 min long. Children were not required to say anything during the task. However, some of the children pointed to the pictures, asked questions, added comments, or responded to parents' questions during the storytelling task. Parents' stories were audio recorded for later transcription and coding analysis.

### Language Sample Transcription

The audio files for each parent-child storytelling task were transcribed by trained research assistants who were native Cantonese-speakers and analyzed using the Systematic Analysis of Language Transcripts Software program (SALT; Miller and Iglesias, 2018). The Cantonese language sample transcription process followed guidelines developed by Klee et al. (2004) and is consistent with previous studies (see Kan et al., 2020). There are three main stages of the transcription process: (1) transcribing narrative recordings in Cantonese into Chinese characters, (2) converting the language samples into Romanized form, and (3) identifying compound words. Each Chinese character carries a specific meaning that can stand on its own or can be combined to create a compound word with a new meaning. For example, in the compound word 開心*hoi1sam1*, individually *hoi1* means “open” and *sam1* means “heart,” but when combined *hoi1sam1* is the word, “happy.” When identifying the compound words, we combined some of the single Chinese characters to create compound words.

Table 2 presents a summary description of parents' Mean Length Utterance (MLU), Number of Different Words (NDW), and Type Token Ratio (TTR) from their storytelling tasks. MLU was calculated by the total number of compound words and single Chinese characters divided by the total number of utterances in the language sample. NDW refers to the number of different words parents used in the storytelling task. TTR was calculated by the NDW divided by the total number of words that parents used in each storytelling task. There were no significant differences in NDW and TTR across books, indicating that all three books elicited similar number of different words from parents. However, parents produced significantly greater MLU in the book, “There's a Witch Under the Stairs” compared to the other two books (*F* = 7.33, *p* < 0.01), suggesting that this book may have offered more opportunities for descriptive sentences than the other ones.

**Table 2:** Summary of parents' language measures (mean and standard deviation) in the storytelling tasks in Cantonese.

	**Alexander book**	**Witch book**	**Llama book**	***F*_**(1, 15)**_**
Mean Length Utterance	5.07 (0.49)	5.69 (1.09)	5.09 (0.62)	7.33^**^
Number of Different Words	154.94 (36.18)	140.63 (35.64)	162.25 (46.79)	1.73
Type Token Ratio	0.24 (0.50)	0.24 (0.04)	0.22 (0.08)	0.78

*p < 0.05,

** *p < 0.01*.

### Coding Emotion Words

The total number of emotion words and total number of different emotion words that parents used during each storytelling task were hand-coded in the language samples. In this study, emotion words were defined as those that directly refer to specific affective states (e.g., happy, angry) or processes (e.g., to worry, to rage), and typically fit in the sentence context, “I am…” or “I feel…” This definition for emotion words is based on the framework developed by Pavlenko (2008) and is consistent with the coding criteria used in other studies that examined Chinese emotion words (i.e., Lin and Yao, 2016; Ng et al., 2019). A trained native Cantonese-speaking research assistant coded the emotion words. To ensure reliability, the research assistant and the first author coded 6% of the language samples and reached 100% agreement. The two coders discussed what constituted as an emotion word based on the coding criteria. If there was confusion about what constituted an emotion word, we discussed it until we reached agreement. The total number of emotion words was calculated by adding the number of times the parent used an emotion word in the storytelling task.

### Coding Emotion Explanations

Parents' emotion explanations during each storytelling task were also hand-coded in Cantonese language samples. Emotion explanations included statements that provided causal information about an emotion (“Llama feels sad because…” or “Alex was angry when his brothers…”) or questions that asked about emotions (“Why does he feel sad?”). Emotion explanations were also coded if there were two consecutive events that were related but not linked with a causal conjunction (“Kiki is scared. The witch was under the stairs.”). The coding criteria for emotion explanations was based on Bloom and Capatides (1987) and is consistent with the criteria used in previous studies (Cervantes and Callahan, 1998; Aznar and Tenebaum, 2013).

## Result

### Parent Questionnaire on Emotion Language Experiences

Parents reported that Cantonese was the language that parents and their child felt most comfortable using when discussing emotions. For the emotion word checklist, parents selected which emotion words they use with their child. There were five emotion categories on the emotion checklist: happy, sad, angry, guilt, scared. Please see Appendix C in Supplementary Material for the complete list and number of emotion words in each category. Table 3 presents the average proportion of emotion words that parents reported using with their child in each category. On average, the happy emotion category had the highest proportion of different emotion words reported by parents (34%), while the guilt emotion category had the lowest proportion of different emotion words (15%). Parents also rated how frequently they talked about certain emotion words with their child in the last 2 weeks. Pearson's correlation analysis was used to examine the relationships among the reported frequency in which parents talk about emotions with their child. Results showed that happy, angry, scared, and sad emotion words have a significant positive relationship with each of the other emotion words (see Table 4). However, guilt did not correlate with any of the other emotion words in terms of how frequently parents talk about that emotion word with their child.

**Table 3:** Average proportion of emotion words reported in each category in the emotion word checklist on the parent questionnaire.

	**Mean**	***SD***	**Range**
Happy	0.34	0.18	0.11–0.78
Angry	0.23	0.12	0–0.44
Scared	0.20	0.13	0–0.50
Sad	0.19	0.09	0.09–0.36
Guilt	0.15	0.10	0–0.30

**Table 4:** Relationships among how frequently parents talk about emotions with their child.

	**Sad**	**Happy**	**Angry**	**Scared**	**Guilty**
Sad	–	–	–	–	–
Happy	0.60^*^	–	–	–	–
Angry	0.76^**^	0.77^**^	–	–	–
Scared	0.65^**^	0.56^*^	0.72^**^	–	–
Guilty	0.15	−0.12	0.06	0.41	–

* *p < 0.05*,

** *p < 0.01*.

### Parent-Child Emotion Elicitation Storytelling Tasks

Table 5 summarizes parents' performance in the three storytelling tasks in Cantonese. Overall, there were no significant differences in the total number of emotion words [*F*_(1,15)_ = 1.03, *p* > 0.05], different emotion words [*F*_(1,15)_ = 0.76, *p* > 0.05], or emotion explanations [*F*_(1,15)_ = 1.76, *p* > 0.05] across the three books that each elicited a different negative emotion word. In the “Alexander and the Terrible, Horrible, No Good, Very Bad Day” storytelling task, parents used a mean of 8.56 (*SD* = 6.80) emotion words, 2.13 (*SD* = 1.09) different emotion words, and 2.00 (*SD* = 3.27) emotion explanations. In the “There's a Witch Under the Stairs” storytelling task, parents used a mean of 8.13 (*SD* = 7.49) emotion words, 1.88 (*SD* =1.41) different emotion words, and 1.06 (*SD* = 1.61) emotion explanations. In the “Llama, Llama Misses Mama” storytelling task, parents used a mean of 11.31 (*SD* = 10.78) emotion words, 2.38 (*SD* = 1.63) different emotion words, and 3.38 (*SD* = 5.5) emotion explanations.

**Table 5:** Parents' performance (mean and standard deviations) in the storytelling tasks in Cantonese.

	**Alexander book**	**Witch book**	**llama book**	***F*_**(1, 15)**_**
Total emotion words	8.56 (6.80)	8.13 (7.49)	11.31 (10.78)	1.03
Different emotion words	2.13 (1.09)	1.88 (1.41)	2.38 (1.63)	0.76
Emotion explanations	2.00 (3.27)	1.06 (1.61)	3.38 (5.5)	1.76

### Comparing Information Between the Parent Questionnaire and Language Samples

In addition to examining the parent questionnaire and the parent language samples individually, our third research question examined whether comparing the two measures would reveal consistencies or differences in the emotion words parents used and reported. For example, did parents who reported using certain emotion words on the questionnaire checklist consistently use those emotion words in the story task? Are there certain emotion words that parents are not likely to report on a questionnaire, but will use when given the chance in a story context? Emotion words that were coded in the language samples were matched against those that parents selected on the questionnaire checklist. Simple likelihood ratio calculations comparing parents' responses on the questionnaire with their performance on the storytelling tasks are shown in Table 6. Additional qualitative analysis examined the types of emotion words parents frequently reported and used.

**Table 6:** A 2 × 2 table comparing parents' responses on the questionnaire with their performance during the storytelling task.

		**Responses on the parent questionnaire**	
		**Reported (%)**	**Did not report (%)**
**Performance on the**	**Used**	5.24	3.90
**storytelling task**	**Did not use**	17.07	73.78

*The table presents the average percentage of emotion words across participants*.

On the questionnaire checklist, parents selected an average of 11.13 (*SD* = 5.00) different emotion words out of the 51 available emotion words. During the storytelling task, 5.24% of parents consistently used the same emotion words they reported on the questionnaire. Those emotion words included *hoi1sam1* (happy), *m4hoi1sam1* (sad), *nau1* (mad), and *ging1* (scared). On average, 3.90% of parents used certain emotion words during the storytelling task but did not report those emotion words on the questionnaire. For example, parents often used the emotion word *faan4* (annoyed/bothered) but did not report this word on the questionnaire. We offer some excerpts from the language samples below to illustrate the words used in the story contexts. Additionally, 17.07% of parents reported emotion words on the questionnaire, but they did not use those emotion words during the storytelling task. For example, some parents reported on the questionnaire that they use emotion words such as *gik7hei3* (furious) or *soeng1sam1* (broken hearted), but they did not use them during the storytelling task. The remaining 73.78% of parents did not report using the emotion words on the questionnaire and did not use those emotion words in the storytelling task. Those emotion words included *geng1tsing1* (scared pale), *gik7sei2jan4* (angry to death), and *faai3lok6* (joy). We will explain possible reasons why parents may consistently use or not use emotion words in the discussion.

***Reported on the Questionnaire and Used in the Story***
Example 1: Ni1 loeng5 go3 siu2 pang4jau5 hai2 dou6 waan2 bo1 bo1. Hou2 *hoi1sam1*.
These two children here are playing ball. Very *happy*.
Example 2: Joeng4 joeng4, nei5 dzou6 mat7 je5 aa? Nei5 dzou6 mat7 *m4hoi1sam1* aa?
Llama llama, what are you doing? Why are you *sad (not happy)*?
Example 3: Jau2 hou2 *nau1* hai2 dou6. Jau2 m4 tung4 daa1di5 jau2 m4 tung4 maa1mi5 king1gai2.
And he's so *mad* here. And he's not talking to daddy or mommy.
Example 4: Mou4po4 soeng2 zuk keoi5 go3 goek8 goek8. Keoi5 hou2 *ging1*, hou2 *ging1*.
The witch wants to grab her feet feet. She is so *scared*, so *scared*.
***Did Not Report on the Questionnaire but Used in the Story***
Example 1: Keoi5 jau2 tok3 dzy5 go3 tau4 aa. Nei5 gok3dak7 keoi5 hai6 mei3 hou2 *faan*4 gam2 aa?
He is also holding his head. Don't you think he looks very *bothered*?
Example 2: D bat7 dou3 hou2 lyun3 ak. Gam2 keoi5 hai6 mei3 hou2 *faan4* aa? Dzing2 dou3 lyun3 tsai3 hai6 mei3 aa?
The pens are also a mess. And so he's *bothered* right? He made a mess, right?

## Discussion

The main goal of this study was to examine socialization of emotions in Chinese American immigrant parents of bilingual bicultural preschool children who are exposed to Cantonese (L1) at home since birth, and then learn English (L2) at school. We used a combination of a Chinese parent questionnaire (broad measure) and parents' Cantonese language samples (narrow measure) to gather more holistic information about parents' socialization of emotions in the home environment. The parent questionnaire and language samples were quantitatively and qualitatively analyzed. There were three main findings from the analyses. First, the Chinese parent questionnaire revealed that the parents in our study did not talk about guilt emotions with their children as often as they do with other emotions. Second, results from Cantonese language samples showed that the parents in our study used each type of negative emotion word with similar frequency in a storytelling context. Lastly, qualitative analysis of the parent questionnaire and the language samples revealed patterns in the ways parents report and use emotion words with their child. Findings illustrate how the parent questionnaire and parents' language samples each offer different information that could complement one another to provide a more comprehensive understanding about parents' emotion talk. These findings will be discussed in greater detail below.

### Chinese American Immigrant Parents' Socialization of Guilt Emotions

Different types of guilt emotion words in the Chinese language and the value of guilt emotions in maintaining appropriate social functioning and behaviors contribute to socialization of guilt emotions in Chinese society and culture (e.g., Bedford, 2004; Wong and Tsai, 2007; Yik, 2010). Preliminary evidence from Chinese parents showed that parents engage in frequent interactions about self-conscious emotions like shame early in their child's development (i.e., Fung, 1999). Accordingly, it can be expected that the parents in our study, who immigrated from China and use Chinese as their dominant language, would also use many guilt emotion words and discuss guilt frequently with their child. However, the current study did not find such pattern in parents of bilingual bicultural Chinese-English children. In contrast, data from the Chinese parent questionnaire showed that guilt did not correlate with any of the other emotion words, indicating that parents do not talk about guilt as frequently as they do with other emotion words. Moreover, data on the emotion word checklist showed that parents reported using very few guilt words with their child compared to the other emotion words. There are several possible explanations for our findings.

First, our interpretation of the results is based on a narrow list of guilt emotion words in the parent questionnaire. The emotion checklist included ten guilt emotion words, which is a limited range of words for parents to choose from. It is possible that there are many more guilt emotion words that parents may use with their child, but they were not listed on the checklist or parents did not recall them to add to the checklist (also see Limitations and Future Directions). Moreover, although some of the negative emotion words on the checklist may have been related to guilt processes, they were categorized under other emotion categories. For example, the words *soeng1sam1* (broken hearted) or *faan4* (annoyed/bothered) were categorized under the Sad and Angry categories on the emotion checklist, respectively. This may be a potential reason for the paucity of guilt words that parents reported. Future studies could consider implementing an emotion checklist that includes guilt and guilt-related words. Additionally, our parents are Chinese American immigrants living in America, while previous studies examined guilt and shame emotion words in Taiwanese adults and parents living in Taiwan (i.e., Fung, 1999; Bedford, 2004). Future studies are needed to examine a wider range of guilt emotion words in Chinese American immigrant families.

Another likely explanation is that guilt is part of the self-conscious emotion's family, which is considered a distinct category of emotions that may be learned and experienced differently compared to basic emotions (e.g., Happy, Sad, Angry) (Leary, 2004). Guilt requires a concept of the self to evaluate that one has failed to meet the appropriate or moral standards within a society (Lagattuta and Thompson, 2007; Wong and Tsai, 2007). Since guilt is a self-conscious emotion, it is harder to label in others compared to basic emotions, and so parents may have fewer opportunities to use it with their child. Moreover, the questionnaire only asks parents to recall the past 2 weeks. Parents may not have had many opportunities to explain and label guilt emotion words with their child in the past 2 weeks compared to the other basic emotion words.

Lastly, a distinctive feature of guilt is that it develops later than basic emotions (Tracy and Robins, 2004). Our findings provide only a snapshot of parents' socialization of guilt emotions in early child development. This pattern is likely to change as children get older and develop greater self-concept. Indeed, a study by Ferguson et al. (1991), examining children between seven to 12 years old, found that younger children's understanding of guilt relies on others' reactions more than older children. The children in our study are even younger (i.e., preschool age) and may not have developed the self-awareness and self-evaluation skills yet to fully understand guilt emotions. In turn, it is possible that parents may be more likely to discuss guilt emotions with their children later when they have developed these skills and it is developmentally appropriate. Future studies should examine parents use of guilt emotion words across a much broader age range. Additionally, although parents may not be using many guilt emotion words at this early stage of development, it is possible that parents may be using other types of emotion words that could still inform children about guilt without using guilt emotion words. Future studies should examine parents use of emotion-related words that describe actions related to guilt (e.g., stealing, cheating, lying) and emotion-laden words that evoke a guilty feeling (e.g., jail, failure, punishment) (Pavlenko, 2008).

### Parents' Use of Negative Emotion Words in a Story Context

Our study also examined parents' socialization of different types of negative emotion words that were used in the Cantonese language samples. Each wordless picture book intended to elicit different types of negative emotion words: (1) “Alexander and the Terrible, Horrible, No Good, Very Bad Day” elicited anger, frustration, and annoyance; (2) “There's a Witch Under the Stairs” elicited fear and worry, and (3) “Llama, Llama Misses Mama” elicited sadness and nervousness. We sought to understand whether parents used one type of negative emotion word more often than the others within its story context. Anger is considered disruptive to maintaining group harmony and interpersonal relationships, and so Chinese parents are likely to explain anger more to help their child self-regulate (Wang, 2003; Fivush and Wang, 2005). Therefore, we expected that in the “Alexander and the Terrible, Horrible, No Good, Very Bad Day” book, parents would use more emotion words, emotion explanations, and different emotion words compared to the other books, especially since the main character's bad mood affected others in the story too. In contrast with findings in previous studies (e.g., Fivush and Wang, 2005), parents in this study used emotion words similarly across all three books, even though each elicited different types of negative emotions. The parents in our study are Chinese American immigrants raising bilingual bicultural children in the United States, which is different from previous studies (e.g., Fivush and Wang, 2005) that examined Chinese parents living in and raising their children in China. Chinese immigrant parents are adapting to the host culture and learning English (acculturation) while maintaining the home language and culture (enculturation) (e.g., Tao et al., 2013). Our parents may be orienting to both American and Chinese cultures as they socialize emotions with their children, and this may involve other factors in the home such as English and Cantonese language use, family members, and storytelling activities.

Another possible explanation is that parents may be more likely to use those emotion words similarly with their child when presented with the story contexts and visuals for different negative emotions. Previous work showed that emotion words were less likely to evoke a mental image and access contextual information compared to concrete words (e.g., animal, balloon) (Altarriba et al., 1999; Altarriba and Bauer, 2004), suggesting that parents may have fewer opportunities to use emotion words. Indeed, Fivush and Wang (2005) simply asked parents to recall an emotional event and discuss it with their child, which may be challenging for parents to use emotion words when there are no visuals or structured context to support them. Our study, however, presented a storytelling context along with images to facilitate parents' use of emotion words. Our findings highlight the need to examine the ways in which negative emotion words are embedded in different contexts and the acculturation and enculturation of Chinese American immigrants, which may contribute to how parents socialize emotions with their child.

### Complementary Use of a Chinese Parent Questionnaire and Cantonese Language Samples

Another important question in this study is what do the similarities and differences between the parent questionnaire (broad measure) and the parent language samples (narrow measure) tell us about how parents use Cantonese emotion words with their children. Qualitative analysis of the individual emotion words across these two measures yielded interesting patterns in the ways parents report and use emotion words. The words that parents consistently reported and used were basic emotion words including *hoi1sam1* (happy), *m4hoi1sam1* (not happy, sad), *nau1* (angry), and *ging1* (scared). A characteristic feature of basic emotions is that they have clearly identifiable and distinguishable facial expressions (i.e., Ekman, 1992, 1993), and so parents accurately identified these facial expressions in the images and correctly labeled them while telling the story to their child. In contrast, there were many more emotion words that parents did not report and did not use in the storytelling tasks. Emotion differentiation refers to the ability to perceive and distinguish a full range of emotions and label these emotions using discreet words to describe different levels of intensity (e.g., Barrett, 2004, 2006). It is possible that our parents may have low emotional differentiation, or a limited range of words to describe emotions, and so they use the same words to describe a few emotional states with their child. A more likely explanation is that the children in our study are only preschool age (3–5 years), and so parents may be using only a few basic Cantonese emotion words that they would expect their child to understand at this age. Although there is normed data on the comprehension and production of these English equivalent emotion words in monolingual English-speaking children (i.e., Ridgeway et al., 1985; Baron-Cohen et al., 2010) and monolingual Chinese-speaking children (i.e., Li and Yu, 2015), we do not know the development of emotion words in children who learn both Cantonese and English. More research examining the emotion words that bilingual bicultural children are exposed to in the home may contribute to our understanding of emotion language development in this population.

Findings also showed that parents underestimate and overestimate using certain emotion words with their child, and this may depend on the contextual availability. Parents often used the word *faan4* (annoyed/bothered) in the “Alexander and the Terrible, Horrible, No Good, Very Bad Day” book, but they underreported this word on the questionnaire. Consistent with previous literature (e.g., Altarriba et al., 1999; Altarriba and Bauer, 2004), parents may not easily access the contextual information for emotion words, and so they may underestimate using some words on the questionnaire but may still use these emotion words when the contextual information is available to them. Interestingly, parents also overestimated using certain emotion words on the questionnaire and did not use them in the storytelling task. For example, *soeng1sam1* which means “broken hearted” is a high arousal, negative emotion words that would only be appropriate to use in highly specific contexts such as a passing of a family member or the ending of a long-term relationship. The images in the books and the storylines may not lend itself to elicit these kinds of high arousal specific emotion words. It is possible, however, that parents may still use these kinds of emotion words in the home environment if they have experienced these kinds of events in the past.

### Limitations and Future Directions

There are several limitations to this study. A major limitation of this study is that we have a small sample size (*n* = 16). We cover a small and relatively homogenous group of Chinese American parents and bilingual bicultural children, and so this may not be representative of the entire population. While the findings in this study shed light on Chinese American immigrant parents' socialization of emotions, we need to be cautious when interpreting the findings. Future studies should look at large sample sizes to see if these results still hold.

A second limitation is the emotion word checklist in the Chinese parent questionnaire. The emotion words on the checklist were selected based on previous monolingual English and Chinese norms (i.e., Ridgeway et al., 1985; Baron-Cohen et al., 2010; Li and Yu, 2015), and they may not cover all the emotion words that Cantonese-English bilingual children know. In particular, the guilt emotion category on the checklist represents a narrow list of words that parents may use. There may be other emotion words that are related to guilt but were categorized under a different emotion category on the checklist. Future studies should consider examining other emotion words that are related to guilt for a more comprehensive understanding about how parents discuss guilt with their child in the home.

Additionally, our results that Chinese American parents may not talk about guilt emotion words as frequently as the other basic emotion words should be interpreted with caution. We only examined the relationship between parents and their child, which provides a limited perspective on parents' socialization of guilt emotions. Socialization of emotions involves telling stories around the child and with the child, modeling behaviors, interacting with other adults, conveying beliefs and ways of thinking and feeling, and through this process children begin to internalize emotion norms and expectations (e.g., Miller et al., 2012; Wang, 2013). Since guilt is highly valued in Chinese culture (e.g., Bedford, 2004; Wong and Tsai, 2007; Yik, 2010), it is possible that children may be exposed to guilt emotions through indirect means from other social interactions between parents or other adult family members with the child as a bystander. Future studies should examine other aspects of socialization beyond direct parent-child interactions, including conversations between parents and other family members in the home, to fully understand socialization of guilt in bilingual children in the home context.

A third limitation is that only parents completed the questionnaire which may provide one perspective of the child's exposure to emotion language in the home environment. Since many of the families in our study lived with their grandparents or additional family members, future studies could have other family members complete the questionnaire. This could potentially reveal whether grandparents use different emotion words with the child compared to the parents. Additionally, given that acculturation and enculturation contribute to parents' orientation to American and Chinese culture, future work should add a question about the parents' length of stay in the United States on the questionnaire.

Lastly, the emotion-elicited storytelling tasks only capture a snapshot of parents' use of emotion words from three books, and so we should be careful to generalize these findings. The storytelling task in our study provided a limited and structured context for parents to use different emotion words. Previous studies asked parents to recall an emotional event and discuss the memories with their children (i.e., Fivush and Wang, 2005), which may capture parents use of emotion words in a more open-ended context. Future studies should examine parents' use of emotion words across different tasks (e.g., storytelling, day-long recordings, memory recall, etc.) to capture more accurately which types of emotion words parents use more often and in which contexts.

## Conclusion and Implications

The main goal of this study was to examine Chinese American immigrant parents' socialization of emotions in sequential bilingual children who are exposed to Cantonese (L1) at home and learn English (L2) at school. The second goal was to explore the use of both a Chinese parent questionnaire and parents' Cantonese language samples to gather more holistic information about parents' emotion talk in bilingual bicultural children's home environment. Results from the Chinese parent questionnaire revealed that the parents in our study did not talk about guilt emotions with their children as often as they do with other emotions. These results were surprising given what we know from the literature that guilt has an important role in social functioning in the Chinese culture, and that Chinese parents talk about self-conscious emotions like shame as early as 2.5 years of age (e.g., Fung, 1999; Bedford, 2004; Wong and Tsai, 2007; Yik, 2010). However, guilt emotions require a concept of the self that may not be developed at this age, and so parents may be less likely to use guilt emotion words at this time. Results from the Cantonese language samples showed that parents in our study used each type of negative emotion word with similar frequency in a story context. Again, these results were surprising since previous work showed that Chinese parents living in China discuss angry emotions more often to help their children self-regulate and maintain social harmony (Wang, 2003; Fivush and Wang, 2005). However, the parents in our study are Chinese American immigrants who are raising bilingual bicultural children in the United States, and so our parents' orientation and adaptation to both Chinese and American cultures may influence how they socialize emotions with their children.

Qualitative analysis of the parent questionnaire and the language samples revealed patterns in the ways parents report and use emotion words with their child. Parents consistently reported and used basic emotion words (happy, sad, angry, scared), possibly because basic emotions have an identifiable and distinguishable facial expression. However, there were many emotion words that parents neither reported nor used, which is likely because our children are young and there is a relatively small range of emotion words that parents would use with their child. Findings also showed that parents underestimate and overestimate using certain emotion words with their child, and this may depend on the contextual availability. Taken together, our findings illustrate how the combined use of the parent questionnaire and parents' language samples each offer different information that could complement one another to provide a more comprehensive understanding about parents' use of emotion words.

Although our findings are preliminary, our study raises awareness that Chinese American immigrant parents have unique and interdependent dual cultural experiences which may influence how they socialize emotions in the home with their bilingual bicultural child. A common assumption is that immigrant parents may adopt more Western practices the longer they live in the host culture; however, it may not be that straightforward. Chinese American immigrant parents may continue to preserve traditional Chinese values and socialize their child accordingly while immersed in the mainstream American culture (e.g., Wang, 2013). Immigrant parents' socialization practices are embedded in both Western and Chinese social contexts and may be influenced by cultural changes in each over time. The findings in our study raise more questions about parents' acculturation and enculturation processes and the dynamic nature of culture in shaping children's emotion language learning. It is important to note that immigrant parents' socialization of emotions can help bilingual bicultural children learn emotions in their home language, while they are also learning emotions in English, thereby increasing their opportunities to communicate emotions in more social contexts and maintain a positive social-emotional well-being.

## Data Availability Statement

The raw data supporting the conclusions of this article will be made available by the authors, without undue reservation.

## Ethics Statement

The studies involving human participants were reviewed and approved by University of Colorado Boulder, Institutional Review Board. Written informed consent to participate in this study was provided by the participants' legal guardian/next of kin.

## Author Contributions

All authors listed have made a substantial, direct and intellectual contribution to the work, and approved it for publication.

## Conflict of Interest

The authors declare that the research was conducted in the absence of any commercial or financial relationships that could be construed as a potential conflict of interest.

## Publisher's Note

All claims expressed in this article are solely those of the authors and do not necessarily represent those of their affiliated organizations, or those of the publisher, the editors and the reviewers. Any product that may be evaluated in this article, or claim that may be made by its manufacturer, is not guaranteed or endorsed by the publisher.
