# Supplementary material for: Chinese American Immigrant Parents' Socialization of Emotions in Bilingual Bicultural Preschool Children
Source: Front Psychol. 2021 Jul 30;12:642417. doi: 10.3389/fpsyg.2021.642417 (PMC8362853; doi:10.3389/fpsyg.2021.642417)
Supplement: Supplementary file 1 [file Data_Sheet_1.DOC]

Appendix A. Sample of the questionnaire asking parents to report hours spent on each storytelling activity and the language distribution. English equivalent translation is provided in brackets.

請填寫**每週**進行下述活動時，您對您孩子使用各語言的比例。

[What percentage of each language is your child exposed to during the following activities **per week**?]

| **活動項目**  **[Activity]** | **每週耗時**  **[Hours spent per week]** | **語言環境之各語言百分比**  **[Percentage of each language exposed]** |
| --- | --- | --- |
| 讀報紙或故事書等文章給您的孩子聽時（書，報紙，等等）  [Reading stories out loud to your child (with books, newspapers, etc.)] |  0-5   6-10   11-15   16-20   21-25   26+   在家中沒有進行這項活動  [This activity is not done at home] |  100% 廣東話/臺山話 [Cantonese/Toisan]   80% 廣東話/臺山話; 20% 英文   60% 廣東話/臺山話; 40% 英文   50% 廣東話/臺山話; 50% 英文   40% 廣東話/臺山話; 60% 英文   20% 廣東話/臺山話; 80% 英文   100% 英文 [English]   其他語言 [Other languages]: __________% ________% |
| 說故事給您的孩子聽時 (沒有故事書或報紙等)  [Telling stories out loud to your child (without books or newspapers, etc.)] |  0-5   6-10   11-15   16-20   21-25   26+   在家中沒有進行這項活動  [This activity is not done at home] |  100% 廣東話/臺山話 [Cantonese/Toisan]   80% 廣東話/臺山話; 20% 英文   60% 廣東話/臺山話; 40% 英文   50% 廣東話/臺山話; 50% 英文   40% 廣東話/臺山話; 60% 英文   20% 廣東話/臺山話; 80% 英文   100% 英文 [English]   其他語言 [Other languages]: __________% ________% |
| 與孩子一同收看電視節目或電影時  [Watching t.v. shows or movies] |  0-5   6-10   11-15   16-20   21-25   26+   在家中沒有進行這項活動  [This activity is not done at home] |  100% 廣東話/臺山話[Cantonese/Toisan]   80% 廣東話/臺山話; 20% 英文   60% 廣東話/臺山話; 40% 英文   50% 廣東話/臺山話; 50% 英文   40% 廣東話/臺山話; 60% 英文   20% 廣東話/臺山話; 80% 英文   100% 英文[English]   其他語言[Other languages]: __________% ________% |
