# Supplementary material for: Chinese American Immigrant Parents' Socialization of Emotions in Bilingual Bicultural Preschool Children
Source: Front Psychol. 2021 Jul 30;12:642417. doi: 10.3389/fpsyg.2021.642417 (PMC8362853; doi:10.3389/fpsyg.2021.642417)
Supplement: Supplementary file 2 [file Data_Sheet_2.DOC]

Appendix B. Sample of the questionnaire about the parent and the child’s emotion language preference and the Likert rating scale. English equivalent translation is provided in brackets.

**情緒 [Feelings]**

1. 當您和您的孩子討論情緒，以下哪種語言**您**使用地最自然？

[Does your child recognize the following feelings? (e.g., in books, people, tv shows?)]

 開心 [Happy]  唔開心 [Sad]  憤怒 [Angry]  驚訝 [Surprised]  驚 [Scared]  內疚 [Guilt]

1. 當您和您的孩子討論情緒，以下哪種語言**您**使用地最自然？

[Which language do **YOU** feel more comfortable using when discussing feelings with your child?]

 廣東話 [Cantonese]  英文 [English]  臺山話 [Toisanese]  其他 [Other]: _______________

1. 當您的孩子和您討論情緒，以下哪種語言**他／她**使用地最自然？

[Which language does **YOUR CHILD** feel more comfortable using when discussing his/her feelings with you?]

 廣東話 [Cantonese]  英文 [English]  臺山話 [Toisanese]  其他 [Other]: _______________

1. **在過去的兩周內**，請問您和您的孩子經歷過多少次以下這些情況？(每種情況請選擇一個答案)

[In the **past two weeks**, how many times have you experienced this kind of situation with your child? (Please choose one answer in each case)]

|  | **0次**  **[Times]** | **1-2次**  **[Times]** | **3-4次**  **[Times]** | **5次或 更多**  **[Times or more]** | **不適用**  **[N/A]** |
| --- | --- | --- | --- | --- | --- |
| - 當我和我的孩子一起讀書時，**我問了他／她**關於故事人物情緒的問題。例如: “Joe有什麼情緒？”   [When we read books together, **I asked my child** about the character’s feelings. Example: *“How does Joe feel?”*] |  |  |  |  |  |
| - 當我和我的孩子一起讀書時，**他／她問了我**關於故事人物情緒的問題或**評論了**故事人物的情緒。例如: “Joe有什麼情緒？為甚麼Joe有這種情緒？”   [When we read books together, **my child asked me** **questions or made comments** about the character’s feelings. Example: *“What does Joe feel? Why does Joe feel that?”*] |  |  |  |  |  |
| - 當我和我的孩子一起讀書時，我回答了他／她關於故事人物情緒的問題。例如: “Joe好傷心，因為他失去了他的狗仔。”   [When we read books together, **I answered my child’s** questions about the character’s feelings. Example: *“Joe feels sad because he lost his dog.”*] |  |  |  |  |  |
| - 當我的孩子問我某人為甚麼傷心，我們討論了那人傷心的原因。   [When my child asked me questions about someone being **sad**, we talked about why that person was sad.] |  |  |  |  |  |
| - 當我的孩子問我某人為甚麼開心，我們討論了那人開心的原因。   [When my child asked me questions about someone being **happy**, we talked about why that person was happy.] |  |  |  |  |  |
| - 當我的孩子問我某人為甚麼憤怒，我們討論了那人憤怒的原因。   [When my child asked me questions about someone being **angry**, we talked about why that person was angry.] |  |  |  |  |  |
| - 當我的孩子問我某人為甚麼害怕，我們 討論了那人害怕的原因。   [When my child asked me questions about someone being **scared**, we talked about why that person was scared.] |  |  |  |  |  |
| - 當我的孩子問我某人為甚麼內疚，我們討論了那人內疚的原因。   [When my child asked me questions about someone being **guilty**, we talked about why that person was guilty.] |  |  |  |  |  |
