# Supplementary material for: Chinese American Immigrant Parents' Socialization of Emotions in Bilingual Bicultural Preschool Children
Source: Front Psychol. 2021 Jul 30;12:642417. doi: 10.3389/fpsyg.2021.642417 (PMC8362853; doi:10.3389/fpsyg.2021.642417)
Supplement: Supplementary file 3 [file Data_Sheet_3.DOC]

Appendix C. The emotion checklist from the parent questionnaire. English equivalent translation is provided in brackets.

當您和您的孩子談論情緒，您使用以下哪些詞彙？

[What words do you use when talking to your child about feelings?]

| **開心 [Happy]** | **唔開心 [Sad/ Not Happy]** | **嬲 [Mad]** |
| --- | --- | --- |
| - 開心 [happy] - 高興 [joyful] - 快樂 [happy for birthdays or new year] - 幸福 [bless/bliss] - 興奮 [excited] - 激動 [excited] - 自豪 [proud]    平靜 [peaceful]   驚訝 [surprised]  Other: ______________________ |  唔開心 [sad/not happy]   傷心 [broken hearted]   難過 [sorrow]   難受 [not well]   灰心 [discouraged]   洩氣 [disappointed]   絕望 [helpless/despair]   閉翳 [depressed]   無助 [helpless]   慘 [miserable]   淒涼 [desolate]  Other: ______________________ |  嬲 [mad]   憤怒 [angry]   火滾 [boiling anger]   穀氣 [built-up anger]   激氣 [furious/agitated]   激死人 [angry to death]   沮喪 [frustrated]   煩 [annoyed/bothered]   妒忌 [jealous]  Other: ______________________ |
| **內疚 [Guilty]** | **害怕 [Scared]** |  |
|  內疚 [guilty]   羞恥 [shame]   慚愧 [ashamed]   無面 [loss of face]   羞家 [family disgrace]   尷尬 [embarrassed]   失望 [disappointed]   掃興[disappointed]   怕醜 [shy]   後悔 [regretful]  Other: ______________________ |  害怕 [scared]   擔心 [worried]   驚 [scared/shock]   得人驚 [shocking]   嚇死 [scared to death]   揗揗震 [scared shaking]   驚青 [scared pale]   囉囉攣 [worried/frazzled]   緊張 [nervous]   不安 [disturbed]   震驚 [shock]   心虛 [lack confidence]  Other: ______________________ |  |
